# Nef interferes with development of thymic T cell precursors: differential mechanisms in HIV and SIV

**DOI:** 10.1186/1742-4690-8-S2-P48

**Published:** 2011-10-03

**Authors:** PJ Meuwissen, KK Ariën, I Vandewalle, E Naessens, H Vanderstraeten, T Taghon, G Vanham, OT Fackler, F Kirchhoff, K Saksela, B Verhasselt

**Affiliations:** 1 Ghent University, HIVlab, Department of Clinical Chemistry, Microbiology and Immunology, Ghent University, Ghent, Belgium; 2 Virology unit, Institute of Tropical Medicine, Antwerp, Belgium; 3 Department of Virology, University of Heidelberg, Heidelberg, Gemany; 4 Institute of Virology, University of Ulm, Ulm, Germany; 5 Institute of Medical Technology, University of Tampere, Tampere, Finland

## Background

HIV infection of the thymus results in a decreased output of naive T cells. We previously showed that expression of the Nef protein alone is sufficient to disturb human thymopoiesis. Additional structure-function studies with mutant Nef alleles suggested a role of PAK2 in this process. In this study we evaluated the effect of Nef alleles from different clinical HIV-1, HIV-2 and SIV isolates and Nef mutants which were specifically mutated in the PAK2 interaction surface for their effect on T cell development.

## Materials and methods

Nef alleles from several HIV primary isolates (HIV-1 subtypes and HIV-2) were amplified by PCR from infected PBMCs. The SIV Nef and Nef-PAK2 mutants used in this study have been described before. All Nef alleles were cloned into a retroviral vector expressing Nef and eGFP from a single bicistronic mRNA (NEF-IRES-eGFP). The effect of Nef on thymopoiesis was studied by transducing CD34+ hematopoietic progenitor cells and subsequent fetal thymic organ culture (FTOC). PAK2 association studies were perfomed using *in vitro* kinase activity assays.

## Results

Development of T cell precursors expressing Nef proteins from several subtypes of HIV-1, HIV-2 and SIV is severely impaired. Furthermore, we showed that mutations of F191/F195, that affect the interaction with PAK2 in NA-7 and SF2 Nef respectively, disturb human thymopoiesis in a less dramatic way than wild type HIV-1 Nef alleles. In the case of NA7 F191R, T cell development was not hampered at all. Mutations in SIVmac239 Nef that disrupt the PxxP motif (AxxA, PxxA, AxxP) and mutations that specifically abolish the interaction with PAK2 (H121R, Y221R, double mutant) still hamper human thymopoiesis. Interestingly, SIV Nef mutants that lost the ability to down regulate CD3-TCR no longer disturb human thymopoiesis.

## Conclusion

Interfering with T cell development is a conserved property among primate lentiviral Nef proteins. In the case for HIV-1 but not SIV, Nef disturbs human thymopoiesis by a PAK2 dependent mechanism. Disturbance of thymopoiesis by SIV Nef likely involves the ability to downmodulate CD3.

